# Prognostic impact of metastatic patterns and treatment modalities on overall survival in lung squamous cell carcinoma: A population-based study

**DOI:** 10.1097/MD.0000000000034251

**Published:** 2023-07-21

**Authors:** Lang Qin, Xiangtian Yu, Chuang Xu, Yangchen Liu

**Affiliations:** a Department of Radiotherapy, Taixing Clinical College of Bengbu Medical College, Bengbu, China; b Department of Anesthesiology, The First Affiliated Hospital of Bengbu Medical College, Bengbu, China; c Department of Orthopedics, Taixing Clinical College of Bengbu Medical College, Bengbu, China.

**Keywords:** lung squamous cell carcinoma, metastasis pattern, overall survival, SEER

## Abstract

This study aimed to investigate the impact of distinct metastasis patterns on the overall survival (OS) of individuals diagnosed with organ metastatic lung squamous cell carcinoma (LUSC). OS was calculated using the Kaplan–Meier method, and univariate and multivariate Cox regression analyses were conducted to further assess prognostic factors. A total of 36,025 cases meeting the specified criteria were extracted from the Surveillance, Epidemiology, and End Results database. Among these patients, 30.60% (11,023/36,025) were initially diagnosed at stage IV, and 22.03% (7936/36,025) of these individuals exhibited metastasis in at least 1 organ, including the liver, bone, lung, and brain. Among the 4 types of single metastasis, patients with bone metastasis had the lowest mean OS, at 9.438 months (95% CI: 8.684–10.192). Furthermore, among patients with dual-organ metastases, those with both brain and liver metastases had the shortest mean OS, at 5.523 months (95% CI: 3.762–7.285). Multivariate Cox regression analysis revealed that metastatic site is an independent prognostic factor for OS in patients with single and dual-organ metastases. Chemotherapy was beneficial for patients with single and multiple-organ metastases; although surgery was advantageous for those with single and dual-organ metastases, it did not affect the long-term prognosis of patients with triple organ metastases. Radiotherapy only conferred benefits to patients with single-organ metastasis. LUSC patients exhibit a high incidence of metastasis at the time of initial diagnosis, with significant differences in long-term survival among patients with different patterns of metastasis. Among single-organ metastasis cases, lung metastasis is the most frequent and is associated with the longest mean OS. Regarding treatment options, patients with single-organ metastasis can benefit from chemotherapy, surgery, and radiotherapy, and those with metastasis in 2 organs can benefit from chemotherapy and surgery. Patients with metastasis in more than 2 organs, however, can only benefit from chemotherapy. Understanding the variations in metastasis patterns assists in guiding pretreatment assessments and in determining appropriate therapeutic interventions for LUSC.

## 1. Introduction

Lung cancer is a predominant tumor in terms of both its frequency and mortality, exhibiting a 5-year survival rate below 16%.^[1,2]^ Among lung cancer cases, non-small cell lung cancer (NSCLC) constitutes 85%, with lung squamous cell carcinoma (LUSC) being one of its prevalent histological subtypes and accounting for approximately 25% of NSCLC cases.^[3]^ LUSC patients typically receive a diagnosis at an older age^[4]^ and exhibit a higher prevalence of chronic obstructive pulmonary disease and pulmonary heart disease.^[5]^ Moreover, LUSC commonly originates in the proximal bronchi and has the propensity to invade major blood vessels,^[6]^ thereby posing challenges for surgical intervention. These distinctive attributes contribute to the limited therapeutic options available for metastatic LUSC, resulting in a median survival period that is approximately 30% shorter than that of other NSCLC subtypes.^[7]^

Distant metastasis constitutes the primary cause of unfavorable treatment outcomes and patient mortality, with approximately 1 to 3rd of LUSC patients reported to exhibit distant metastasis upon diagnosis.^[8]^ Consequently, the 5-year survival rate for these patients is below 5%.^[9]^ Metastasis most frequently occurs in the brain, bones, distant lymph nodes, and liver.^[10,11]^ The process of lung cancer metastasis is remarkably intricate, involving interplay between the tumor microenvironment and cancer stem cells.^[12–14]^ In recent years, numerous novel drugs to treat metastatic NSCLC have been explored in clinical trials, affirming their efficacy and expanding the range of first-line treatment options available to patients. The advent of innovative therapies, encompassing targeted therapies and immunotherapies, has led to promising outcomes in prolonging patient survival. Nevertheless, the treatment landscape for metastatic LUSC remains challenging due to the scarcity of mutations in targets such as EGFR,^[15]^ as well as the emergence of resistance and intricate toxic reactions among certain patients following an extended treatment duration.

To date, there have been limited comprehensive retrospective investigations conducted on metastatic LUSC. Therefore, this study utilized the extensive Surveillance, Epidemiology, and End Results (SEER) database to explore the occurrence and distribution of distant organ metastasis in LUSC and to evaluate the influence of diverse treatment approaches on the long-term prognosis of patients. The primary objective of this study was to enhance our comprehension regarding prognosis associated with distinct patterns of organ metastasis, furnishing valuable insights to guide individualized treatment strategies.

## 2. Methods and materials

### 2.1. Patient selection

The present study employed a retrospective cohort study design by utilizing the SEER database of the National Cancer Institute (http://seer.cancer.gov/) to investigate patients with LUSC staged according to the 7th edition of the American Joint Committee on Cancer TNM classification system between 2010 and 2015. The inclusion criterion was LUSC diagnosis (ICD-O-3: 8070–8078) between 2010 and 2015. Exclusion criteria were as follows: Age below 18 years old; Missing data on age, race, sex, marital status, distant metastasis information, tumor grade, primary site, T stage, N stage, surgical information, radiation information, or chemotherapy information; Nonprimary tumors; Survival time < 1 month. This study collected the demographic characteristics of patients, including age, race, sex, and marital status; clinical and pathological characteristics, including tumor grade, primary site, T stage, N stage, bone metastasis, lung metastasis, liver metastasis, and brain metastasis; treatment information, including surgery, radiation therapy, and chemotherapy; and follow-up data, including survival status and overall survival (OS), as defined as the time from diagnosis to death from any cause or the end of follow-up. Lung metastasis is defined in the SEER database as the presence of the tumor nodule in the contralateral lung lobe.

### 2.2. Statistical analysis

Pearson’s chi-squared test was used to analyze differences in the distribution of categorical variables between groups with and without metastasis. The odds ratio (OR) of specific-site metastasis was calculated using logistic regression. The Kaplan–Meier (KM) method was used to generate survival curves for different groups and the log-rank test to determine differences between groups. Univariate and multivariate Cox regression was used to evaluate associations between different variables and prognosis. All data analyses were performed using R statistical software (version 4.2.1, http://www.R-project.org). The “tableone” and “rio” packages were used for chi-squared tests and data output. The “survival,” “survminer,” and “plyr” packages were used for the KM method, log-rank test, logistic regression, and Cox regression analyses. The significance level was set at *P* < .05.

### 2.3. Ethical statement

The SEER database is a public database, and ethical approval has been obtained for the patients included in the database. Users can download relevant data for free for research and publish relevant articles. As our study is based on open-source data, there are no ethical issues or other conflicts of interest.

## 3. Results

### 3.1. Patient characteristics and metastasis patterns

A total of 36,025 LUSC patients were included in the study, of whom 30.60% (11,023/36,025) were diagnosed with stage IV disease at their initial visit; 22.03% (7936/36,025) had metastasis in at least 1 organ, including the liver, bone, lung, and brain. Specifically, 9.28% (3343) had bone metastasis, 5.05% (1821) had brain metastasis, 10.04% (3618) had lung metastasis, and 5.03% (1811) had liver metastasis. Table 1 summarizes the baseline characteristics of all patients. Patients under 65 years old had a higher incidence of bone, brain, and liver metastasis (*P* < .05); male patients had a higher incidence of bone, lung, and liver metastasis (*P* < .05), and unmarried patients had a higher incidence of lung metastasis (*P* < .05). In addition, significant differences (*P* < .05) were observed between metastatic and nonmetastatic groups in terms of race, tumor grade, primary site, T stage, N stage, and treatment modality.

**Table 1:** Clinical features and metastasis sites.

Features	Bone metastasis		*P* value	Brain metastasis		*P* value	Lung metastasis		*P* value	Liver metastasis		*P* value
	No	Yes		No	Yes		No	Yes		No	Yes	
Age			<.001			<.001			.058			<.001
<65	9413 (28.77%)	1266 (36.35%)		9908 (28.82%)	771 (42.34%)		9565 (29.34%)	1114 (30.88%)		10,020 (29.13%)	659 (36.39%)	
≥65	23,309 (71.23%)	2217 (63.65%)		24,476 (71.18%)	1050 (57.66%)		23,032 (70.66%)	2494 (69.12%)		24,374 (70.87%)	1152 (63.61%)	
Race												
White	27,298 (83.42%)	2743 (78.75%)	<.001	28,586 (83.14%)	1455 (79.90%)	.001	27,197 (83.43%)	2844 (78.82%)	<.001	28,605 (83.17%)	1436 (79.29%)	<.001
Black	3798 (11.61%)	513 (14.73%)		4045 (11.76%)	266 (14.61%)		3770 (11.57%)	541 (14.99%)		4036 (11.73%)	275 (15.18%)	
Others	1626 (4.97%)	227 (6.52%)		1753 (5.10%)	100 (5.49%)		1630 (5.00%)	223 (6.18%)		1753 (5.10%)	100 (5.52%)	
Sex			<.001			.437			.039			<.001
Female	12,533 (38.30%)	1089 (31.27%)		12,953 (37.67%)	669 (36.74%)		12,322 (37.80%)	1300 (36.03%)		13,084 (38.04%)	538 (29.71%)	
Male	20,189 (61.70%)	2394 (68.73%)		21,431 (62.33%)	1152 (63.26%)		20,275 (62.20%)	2308 (63.97%)		21,310 (61.96%)	1273 (70.29%)	
Marriage			.052			.311			.016			.524
Married	16,772 (51.26%)	1846 (53.00%)		17,660 (51.36%)	958 (52.61%)		16,832 (51.64%)	1786 (49.50%)		17,673 (51.38%)	945 (52.18%)	
Unmarried	15,950 (48.74%)	1637 (47.00%)		16,724 (48.64%)	863 (47.39%)		15,765 (48.36%)	1822 (50.50%)		16,721 (48.62%)	866 (47.82%)	
Grade			<.001			<.001			<.001			<.001
Well	669 (2.04%)	44 (1.26%)		700 (2.04%)	13 (0.71%)		664 (2.04%)	49 (1.36%)		703 (2.04%)	10 (0.55%)	
Moderate	9775 (29.87%)	519 (14.90%)		10,019 (29.14%)	275 (15.10%)		9584 (29.40%)	710 (19.68%)		10,038 (29.19%)	256 (14.14%)	
Poorly	11,551 (35.30%)	1111 (31.90%)		12,029 (34.98%)	633 (34.76%)		11,470 (35.19%)	1192 (33.04%)		12,072 (35.10%)	590 (32.58%)	
Undifferentiated	187 (0.57%)	21 (0.60%)		195 (0.57%)	13 (0.71%)		189 (0.58%)	19 (0.53%)		202 (0.59%)	6 (0.33%)	
Unknown	10,540 (32.21%)	1788 (51.34%)		11,441 (33.27%)	887 (48.71%)		10,690 (32.79%)	1638 (45.40%)		11,379 (33.08%)	949 (52.40%)	
Primary site			<.001			<.001			<.001			<.001
Main bronchus	1805 (5.52%)	241 (6.92%)		1921 (5.59%)	125 (6.86%)		1799 (5.52%)	247 (6.85%)		1915 (5.57%)	131 (7.23%)	
Upper	18,335 (56.03%)	1830 (52.54%)		19,172 (55.76%)	993 (54.53%)		18,354 (56.31%)	1811 (50.19%)		19,263 (56.01%)	902 (49.81%)	
Middle	9570 (29.25%)	964 (27.68%)		10,050 (29.23%)	484 (26.58%)		9545 (29.28%)	989 (27.41%)		10,026 (29.15%)	508 (28.05%)	
Lower	1195 (3.65%)	120 (3.45%)		1239 (3.60%)	76 (4.17%)		1203 (3.69%)	112 (3.10%)		1241 (3.61%)	74 (4.09%)	
Others	1817 (5.55%)	328 (9.42%)		2002 (5.82%)	143 (7.85%)		1696 (5.20%)	449 (12.44%)		1949 (5.67%)	196 (10.82%)	
T stage			<.001			<.001			<.001			<.001
T1	6579 (20.11%)	237 (6.80%)		6682 (19.43%)	134 (7.36%)		6690 (20.52%)	126 (3.49%)		6700 (19.48%)	116 (6.41%)	
T2	11,502 (35.15%)	958 (27.51%)		11,882 (34.56%)	578 (31.74%)		11,934 (36.61%)	526 (14.58%)		11,941 (34.72%)	519 (28.66%)	
T3	7597 (23.22%)	1033 (29.66%)		8133 (23.65%)	497 (27.29%)		7535 (23.12%)	1095 (30.35%)		8101 (23.55%)	529 (29.21%)	
T4	7044 (21.53%)	1255 (36.03%)		7687 (22.36%)	612 (33.61%)		6438 (19.75%)	1861 (51.58%)		7652 (22.25%)	647 (35.73%)	
N stage			<.001			<.001			<.001			<.001
N0	15,466 (47.26%)	730 (20.96%)		15,819 (46.01%)	377 (20.70%)		15,448 (47.39%)	748 (20.73%)		15,838 (46.05%)	358 (19.77%)	
N1	3427 (10.47%)	328 (9.42%)		3578 (10.41%)	177 (9.72%)		3485 (10.69%)	270 (7.48%)		3591 (10.44%)	164 (9.06%)	
N2	10,700 (32.70%)	1744 (50.07%)		11,506 (33.46%)	938 (51.51%)		10,780 (33.07%)	1664 (46.12%)		11,509 (33.46%)	935 (51.63%)	
N3	3129 (9.56%)	681 (19.55%)		3481 (10.12%)	329 (18.07%)		2884 (8.85%)	926 (25.67%)		3456 (10.05%)	354 (19.55%)	
Surgery			<.001			<.001			<.001			<.001
No	22,896 (69.97%)	3422 (98.25%)		24,566 (71.45%)	1752 (96.21%)		22,826 (70.02%)	3492 (96.78%)		24,544 (71.36%)	1774 (97.96%)	
Yes	9826 (30.03%)	61 (1.75%)		9818 (28.55%)	69 (3.79%)		9771 (29.98%)	116 (3.22%)		9850 (28.64%)	37 (2.04%)	
Radiation			<.001			<.001			<.001			<.001
No	15,961 (48.78%)	1346 (38.64%)		16,941 (49.27%)	366 (20.10%)		15,288 (46.90%)	2019 (55.96%)		16,258 (47.27%)	1049 (57.92%)	
Yes	16,761 (51.22%)	2137 (61.36%)		17,443 (50.73%)	1455 (79.90%)		17,309 (53.10%)	1589 (44.04%)		18,136 (52.73%)	762 (42.08%)	
Chemotherapy			<.001			<.001			<.001			<.001
No	17,388 (53.14%)	1567 (44.99%)		18,136 (52.75%)	819 (44.98%)		17,332 (53.17%)	1623 (44.98%)		18,176 (52.85%)	779 (43.01%)	
Yes	15,334 (46.86%)	1916 (55.01%)		16,248 (47.25%)	1002 (55.02%)		15,265 (46.83%)	1985 (55.02%)		16,218 (47.15%)	1032 (56.99%)	

The metastatic patterns of LUSC are presented in Table 2. Among the 4 potential single-organ metastases, lung metastasis was most prevalent (6.33%), followed by bone metastasis (5.09%) and brain metastasis (2.66%); liver metastasis was least frequent (1.77%). Among the 6 possible combinations of dual-organ metastases, bone and lung metastases were most common (1.36%), whereas brain and liver metastases were least common (0.28%). Among the 4 potential combinations of triple organ metastases, bone, liver, and lung metastases were most prevalent (0.54%), and brain, liver, and lung metastases were least common (0.14%). Four-organ metastases were observed in 0.22% of all patients. Logistic regression analysis (Table 3) further revealed that patients with liver metastasis were more likely to have bone metastasis (OR: 9.756, 95% CI: 8.822–10.789, *P* < .001). Additionally, patients with brain metastasis (OR: 4.767, 95% CI: 4.182–5.434, *P* < .001) and lung metastasis (OR: 4.159, 95% CI: 3.733–4.633, *P* < .001) had a higher likelihood of having liver metastasis.

**Table 2:** Frequencies of combination metastasis.

Features	Lung squamous cell carcinoma	
	Number	Percentage (%)
One site		
Only bone	1842	5.09
Only brain	964	2.66
Only liver	642	1.77
Only lung	2293	6.33
Two sites		
Bone and brain	237	0.65
Bone and liver	449	1.24
Bone and lung	492	1.36
Brain and liver	101	0.28
Brain and lung	201	0.56
Liver and lung	201	0.56
Three sites		
Bone and brain and liver	93	0.26
Bone and brain and lung	96	0.27
Bone and liver and lung	196	0.54
Brain and liver and lung	51	0.14
Four sites		
Bone and brain and liver and lung	78	0.22

**Table 3:** Odds ratio (OR) comparison of different dual-organ metastasis combinations.

Features	Bone metastasis	*P* value	Brain metastasis	*P* value	Lung metastasis	*P* value	Liver metastasis	*P* value
	OR (95% CI)		OR (95% CI)		OR (95% CI)		OR (95% CI)	
Metastasis site								
Bone	1		4.034 (3.617–4.500)	<.001	3.590 (3.293–3.914)	<.001	9.756 (8.822–10.789)	<.001
Brain	4.034 (3.617–4.500)	<.001	1		2.994 (2.671–3.358)	<.001	4.767 (4.182–5.434)	<.001
Lung	3.590 (3.293–3.914)	<.001	2.994 (2.671–3.358)	<.001	1		4.159 (3.733–4.633)	<.001
Liver	9.756 (8.822–10.789)	<.001	4.767 (4.182–5.434)	<.001	4.159 (3.733–4.633)	<.001	1	

### 3.2. Survival analysis of single-organ metastasis

**Figure 1.**
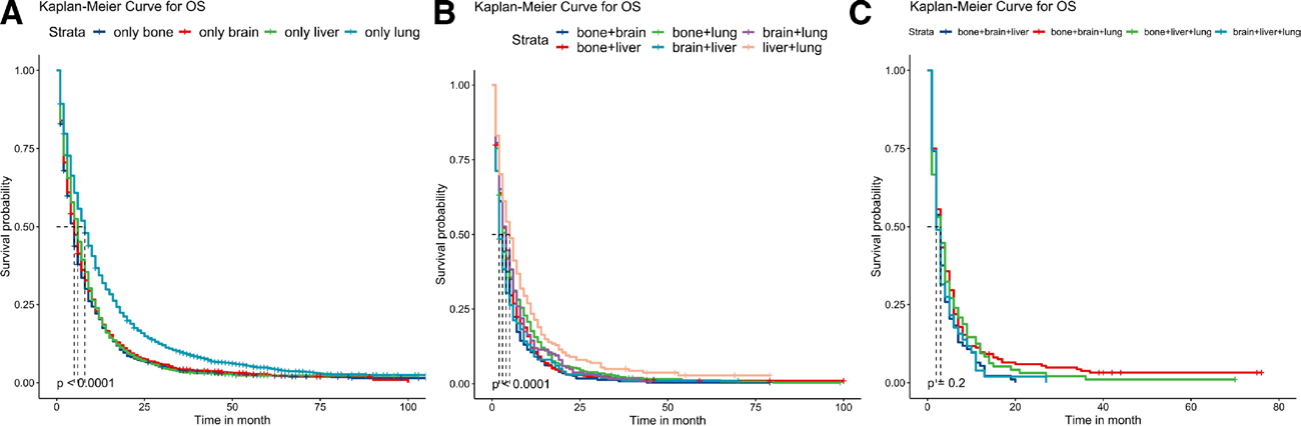
Kaplan–Meier survival analyses to estimate OS for patients with single-organ metastases (A), dual-organ metastases (B), and triple organ metastases (C). OS = overall survival.

**Figure 2.**
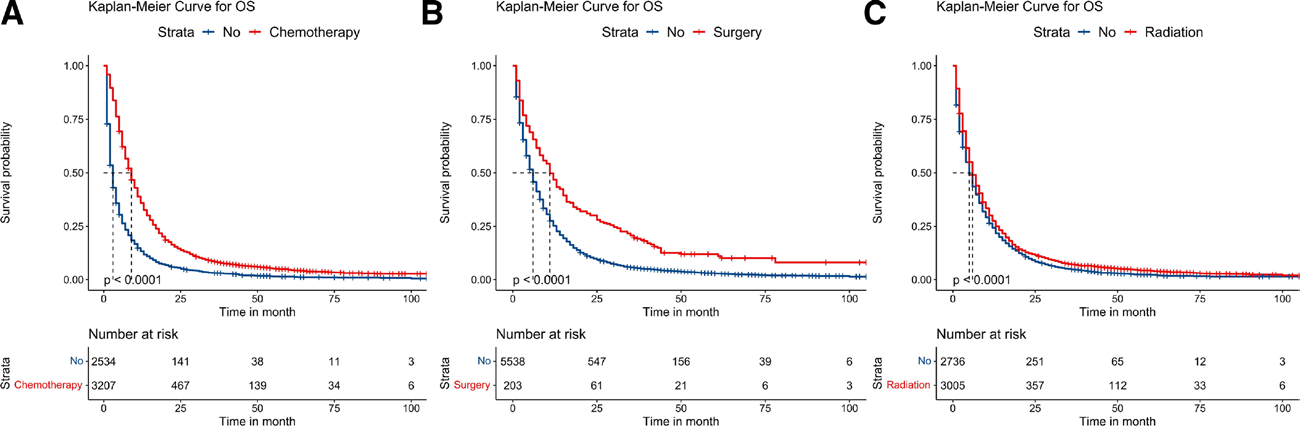
Kaplan–Meier survival analyses to estimate chemotherapy (A), surgery (B), and radiation (C) for OS in patients with single-organ metastasis. OS = overall survival.

In univariate analysis (Table 4), we observed significant impacts of metastatic site, age, sex, marital status, N stage, surgery, radiation therapy, and chemotherapy on the OS of patients with single-organ metastasis (*P* < .05). Notably, patients with lung metastasis exhibited the longest mean OS of 14.819 months (95% CI: 13.955–15.683), whereas those with bone metastasis had the shortest mean OS of 9.438 months (95% CI: 8.684–10.192). To enhance understanding of survival differences, we constructed KM survival curves (Fig. 1A). Our findings revealed that radiation therapy, chemotherapy, and surgery were significant protective factors (*P* < .05), as demonstrated by the survival curves shown in Figure 2. Subsequently, variables demonstrating statistical significance in univariate analysis were included in multivariate Cox regression analysis (Table 5). The results indicated that metastatic site, age, sex, marital status, N stage, surgery, radiation therapy, and chemotherapy were independent prognostic factors for OS (*P* < .05). Among the various metastatic organs, bone (HR: 1.628, 95% CI: 1.525–1.737, *P* < .001), liver (HR: 1.441, 95% CI: 1.316–1.578, *P* < .001), and brain (HR: 1.675, 95% CI: 1.543–1.819, *P* < .001) metastasis carried higher risk than lung metastasis.

**Table 4:** Univariate survival analysis of patients with four single metastases.

Risk factors	Mean of survival months	95% CI	*P* value
Metastasis site			<.001
Bone metastasis	9.438	8.684–10.192	
Brain metastasis	9.876	8.861–10.891	
Liver metastasis	10.094	8.820–11.368	
Lung metastasis	14.819	13.955–15.683	
Age			<.001
<65	13.437	12.485–14.388	
≥65	10.925	10.376–11.475	
Race			.185
White	11.575	11.049–12.101	
Black	11.721	10.406–13.037	
Others	13.213	11.134–15.291	
Sex			.005
Female	12.645	11.763–13.527	
Male	11.183	10.620–11.746	
Marriage			<.001
Married	12.692	11.968–13.416	
Unmarried	10.721	10.090–11.352	
Grade			.187
Well	12.138	9.185–15.090	
Moderate	11.962	10.940–12.983	
Poorly	12.047	11.168–12.925	
Undifferentiated	9.703	6.000–13.405	
Unknown	11.287	10.602–11.972	
Primary site			.091
Main bronchus	9.702	8.232–11.172	
Upper	11.766	11.094–12.438	
Middle	12.645	9.998–15.291	
Lower	11.501	10.657–12.346	
Others	12.847	11.063–14.632	
T stage			.127
T1	13.320	11.395–15.245	
T2	11.200	10.335–12.064	
T3	11.741	10.808–12.674	
T4	11.796	11.002–12.589	
N stage			<.001
N0	14.398	13.258–15.538	
N1	12.442	10.737–14.147	
N2	10.182	9.571–10.792	
N3	11.101	10.227–11.975	
Surgery			<.001
No	11.288	10.818–11.758	
Yes	23.253	19.024–27.482	
Radiation			<.001
No	10.446	9.830–11.061	
Yes	12.874	12.148–13.600	
Chemotherapy			<.001
No	7.035	6.521–7.550	
Yes	15.428	14.690–16.165	

**Table 5:** Multivariate survival analysis of patients with four single metastases.

Risk factors	HR	95% CI	*P* value
Metastasis site			
Bone metastasis	1.628	1.525–1.737	<.001
Brain metastasis	1.675	1.543–1.819	<.001
Liver metastasis	1.441	1.316–1.578	<.001
Lung metastasis	Ref		
Age			
<65	Ref		
≥65	1.137	1.072–1.206	<.001
Sex			
Female	Ref		
Male	1.081	1.021–1.144	.008
Marriage			
Married	Ref		
Unmarried	1.064	1.007–1.123	.027
N stage			
N0	Ref		
N1	1.243	1.122–1.377	<.001
N2	1.431	1.339–1.530	<.001
N3	1.463	1.345–1.590	<.001
Surgery			
No	Ref		
Yes	0.578	0.498–0.672	<.001
Radiation			
No	Ref		
Yes	0.847	0.800–.897	<.001
Chemotherapy			
No	Ref		
Yes	0.467	0.441–0.495	<.001

### 3.3. Survival analysis of multiple-organ metastases

**Figure 3.**
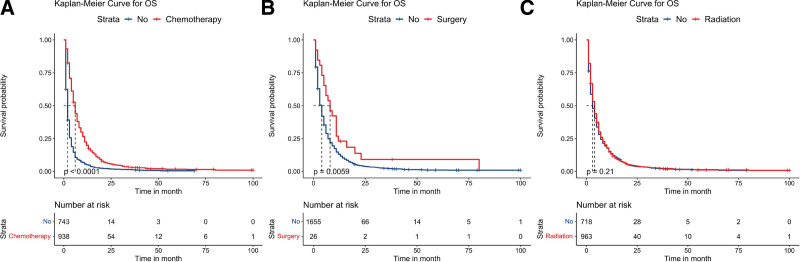
Kaplan–Meier survival analyses to estimate chemotherapy (A), surgery (B), and radiation (C) for OS in patients with dual-organ metastasis. OS = overall survival.

After conducting univariate analysis (see Table S1, Supplemental Digital Content, http://links.lww.com/MD/J261, which illustrates the univariate survival analysis of patients with 2 metastatic sites), we observed that metastatic site, age, T stage, N stage, surgery, and chemotherapy exerted significant influences on the OS of patients with dual-organ metastases (*P* < .05). Specifically, patients with brain and liver metastases exhibited the shortest mean OS of 5.523 months (95% CI: 3.762–7.285); those with lung and liver metastases showed the longest mean OS of 9.882 months (95% CI: 7.826–11.938). We incorporated the variables that displayed statistical significance from univariate analysis into multivariate Cox regression analysis (see Table S2, Supplemental Digital Content, http://links.lww.com/MD/J262, which illustrates the multivariate survival analysis of patients with 2 metastatic sites) and found that metastatic site, T stage, N stage, and chemotherapy were independent prognostic factors for the OS of patients with dual-organ metastases (*P* < .05). Moreover, we summarized the survival curves illustrating different metastasis patterns (Fig. 1B) and the impact of various treatment methods on OS (Fig. 3) within this patient population. Notably, surgery and chemotherapy significantly improved patient OS (*P* < .05), though radiation therapy did not have a statistically significant effect on long-term prognosis (*P* = .21).

**Figure 4.**
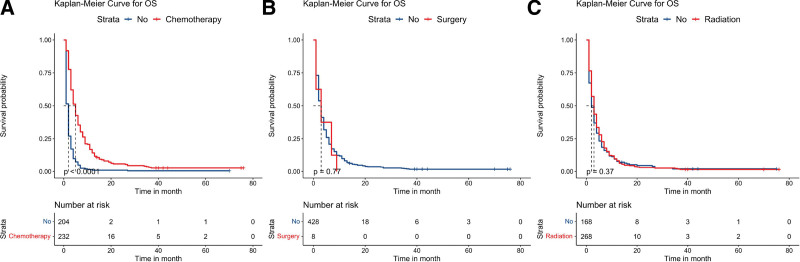
Kaplan–Meier survival analyses to estimate chemotherapy (A), surgery (B), and radiation (C) for OS in patients with triple organ metastasis. OS = overall survival.

Furthermore, we investigated the outcomes of patients with triple-organ metastases. Univariate analysis (see Table S3, Supplemental Digital Content, http://links.lww.com/MD/J263, which illustrates the Univariate survival analysis of patients with 3 metastatic sites) revealed that age and chemotherapy significantly affected the OS of this patient population (*P* < .05). We also summarized the survival curves for different metastasis patterns (Fig. 1C) and the effects of different treatment methods on OS (Fig. 4) for this patient population, with only chemotherapy improving OS (*P* < .05).

## 4. Discussion

The prognosis of lung cancer patients is closely associated with the presence of distant metastasis. Patients with local disease have a 5-year survival rate of 57%, whereas those with metastatic disease have a significantly lower rate of only 5%.^[16]^ LUSC, a common subtype of NSCLC, shows notable distinctions in terms of pathogenesis, patient demographics, metastatic patterns, treatment options, and long-term prognosis compared to other NSCLC subtypes.^[17]^ In our study, we focused on the metastatic patterns and survival prognosis of patients with organ metastatic LUSC while analyzing the value of different treatment modalities for prognosis.

In this study, we found that in LUSC, single-organ metastasis mainly occurs in the lung (6.33%) or bone (5.09%). Notably, patients with lung metastasis exhibited the longest OS, and multivariate Cox analysis further corroborated these findings, demonstrating that patients with lung metastasis had a more favorable prognosis, which aligns with previous research.^[18]^ However, despite the relatively better prognosis associated with lung metastasis compared to metastasis to other organs, we recommend regular monitoring of suspicious lung nodules to mitigate the risk of death due to the high incidence of lung metastasis. Patients with bone metastasis had the worst OS, and Cetin et al^[19]^ study found that bone metastasis is a significant risk factor that affects the prognosis of LUSC patients, significantly impacting their quality of life, increasing their risk of death, and leading to at least 1 skeletal-related event per year. Bisphosphonates, such as zoledronic acid, are commonly used to treat bone metastasis in clinical practice, but their long-term use is limited by severe adverse reactions, such as renal toxicity.^[20]^ Previous studies have shown that NSCLC patients with liver metastasis have the worst prognosis.^[21,22]^ However, our study found that LUSC patients with liver metastasis had a longer mean OS than those with bone or brain metastasis, which may be due to our focus on LUSC over other NSCLC subtypes. It is worth noting that although the proportion of LUSC patients with liver metastasis was lowest among patients with single-organ metastasis (1.77%), the OR of liver metastasis in patients with bone, brain, or lung metastasis was higher than that of other organ metastases, highlighting the need for extra attention to the liver situation of patients with organ metastasis. Liu et al^[23]^ also identified that LUSC patients with brain metastasis faced a greater risk of mortality than those with metastasis to other organs, aligning with our study’s findings. Despite recent advancements in the understanding of lung cancer brain metastasis,^[24,25]^ the specific underlying mechanisms remain unclear. The blood–brain barrier poses a challenge because it restricts full penetration of many drugs into the brain, resulting in poor prognosis for lung cancer patients with brain metastasis. Bevacizumab, an antiangiogenic drug, has demonstrated efficacy in suppressing the proliferation of NSCLC brain metastasis and has emerged as a new treatment option in clinical practice.^[26,27]^

Previous studies have often neglected analysis of metastasis patterns and prognosis in NSCLC patients with multiorgan metastases.^[21,28]^ In our study cohort, we identified 1681 LUSC patients (4.65%) with metastases in 2 organs, a higher occurrence compared to single brain metastasis (2.66%) or liver metastasis (1.77%). Notably, patients with both liver and lung metastases exhibited the longest OS, surpassing even those with single bone or brain metastasis. Conversely, patients with both bone and brain metastases had the poorest prognosis. Furthermore, multivariate Cox regression analysis confirmed that the metastatic site involved independently influenced the prognosis of LUSC patients with dual-organ metastases. However, we observed no significant difference in OS among LUSC patients with triple-organ metastases in univariate analysis, and the number of patients with 4-organ metastases was insufficient for further survival analysis.

Positive driver mutations in lung adenocarcinoma can be targeted with therapy, but there are limited options for LUSC.^[29]^ Historically, platinum-based combination chemotherapy has been the first-line treatment for advanced LUSC. However, recently, first-line immunotherapy-based regimens have emerged as treatment options, with the KEYNOTE-407 trial (NCT02775435) demonstrating that adding pembrolizumab to chemotherapy significantly improves OS and progression-free survival in patients with metastatic LUSC.^[30]^ The latest developments in immunotherapy and targeted therapy have led to significant improvement in survival rates,^[31]^ and the concept of oligometastasis is increasingly mentioned in relation to NSCLC. Oligometastasis can be broadly defined as a limited metastatic tumor burden state with only 1 or a few metastatic sites that may benefit from curative surgery and radiation therapy.^[32,33]^ In our study, we focused on the impact of chemotherapy, radiation therapy, and surgery on the long-term prognosis of organ metastatic LUSC and found that chemotherapy has a significant protective effect on both single and multiple (double or triple) organ metastatic LUSC. Patients with single-organ metastasis who received radiation therapy had better OS than those who did not, even though radiation therapy did not improve the long-term prognosis of patients with multiple-organ metastases. Previous studies have shown that some advanced NSCLC patients with oligometastatic disease can benefit from surgery,^[34]^ and our study confirmed that surgery can benefit patients with single and double organ metastases in LUSC, but with no statistically significant benefit for those with triple-organ metastases. However, the number of patients who underwent surgery in our cohort was small, and further research is needed to clarify the significance.

This study has several limitations that should be noted. First, the study did not include other variables that may affect prognosis, such as immunotherapy, targeted therapy, specific doses and methods of radiotherapy, chemotherapy regimens, tumor markers, and genetic testing results, which limited further analysis. Second, the SEER database contains limited information on organ metastases, including only lung, liver, brain, and bone metastases, even though these are the most common sites of metastasis in LUSC. Third, the limited number of surgical patients and the lack of specific differentiation in surgical approaches may lead to bias.

## 5. Conclusion

LUSC patients exhibit a high incidence of metastasis at initial diagnosis, with metastatic site serving as an independent prognostic factor for single-organ and dual-organ metastasis patients. Among single-organ metastases, lung metastasis was the most common with the longest mean OS, and bone metastasis had the shortest mean OS. Among dual-organ metastases, patients with brain and liver metastases exhibited the shortest mean OS, whereas those with lung and liver metastases showed the longest mean OS. Regarding treatment options, patients with single-organ metastasis can benefit from chemotherapy, surgery, and radiotherapy, and those with metastasis in 2 organs can benefit from chemotherapy and surgery. Patients with metastasis in more than 2 organs, however, can only benefit from chemotherapy. Understanding the variations in metastasis patterns assists in guiding pretreatment assessments and determining appropriate therapeutic interventions for LUSC.

## Author contributions

**Conceptualization:** Yangchen Liu.

**Data curation:** Lang Qin, Chuang Xu, Yangchen Liu.

**Formal analysis:** Chuang Xu.

**Investigation:** Lang Qin.

**Methodology:** Lang Qin.

**Resources:** Xiangtian Yu.

**Software:** Lang Qin.

**Supervision:** Xiangtian Yu.

**Validation:** Xiangtian Yu.

**Visualization:** Xiangtian Yu, Chuang Xu.

**Writing – original draft:** Lang Qin.

**Writing – review & editing:** Yangchen Liu.

## Supplementary Material
