# Supplementary material for: Prognostic impact of metastatic patterns and treatment modalities on overall survival in lung squamous cell carcinoma: A population-based study
Source: Medicine (Baltimore). 2023 Jul 21;102(29):e34251. doi: 10.1097/MD.0000000000034251 (PMC10662909; doi:10.1097/MD.0000000000034251)
Supplement: Supplementary file 1 [file medi-102-e34251-s001.pdf]

Table S1 Univariate survival analysis of patients with two metastatic sites.

Risk factors	Mean of survival months	95% CI	p
**Metastasis site**			<0.001
Bone and brain metastasis	5.489	4.505-6.473	
Bone and liver metastasis	6.591	5.528-7.655	
Bone and lung metastasis	7.302	6.280-8.323	
Brain and liver metastasis	5.523	3.762-7.285	
Brain and lung metastasis	6.564	5.479-7.648	
Liver and lung metastasis	9.882	7.826-11.938	
**Age**			0.001
＜65	7.913	6.967-8.858	
⩾65	6.393	5.764-7.022	
**Race**			0.519
White	6.976	6.408-7.545	
Black	7.538	5.762-9.314	
Others	5.537	4.547-6.528	
**Sex**			0.285
Female	7.341	6.367-8.316	
Male	6.816	6.171-7.461	
**Marriage**			0.256
Married	7.228	6.430-8.026	
Unmarried	6.863	6.061-7.664	
**Grade**			0.871
Well	4.333	1.761-6.906	
Moderate	7.339	5.887-8.790	
Poorly	6.976	6.055-7.898	
Undifferentiated	5.875	3.491-8.259	
Unknown	6.902	6.165-7.638	
**Primary Site**			0.361
Main bronchus	5.441	4.439-6.443	
Upper	7.678	6.765-8.591	
Middle	7.248	5.183-9.313	
Lower	6.478	5.565-7.392	
Others	6.238	5.287-7.188	
**T stage**			0.026
T1	10.533	7.093-13.973	
T2	7.251	6.086-8.416	
T3	6.647	5.717-7.577	
T4	6.624	5.867-7.381	
**N stage**			0.040
N0	8.528	6.724-10.332	
N1	6.928	5.174-8.682	
N2	6.335	5.703-6.967	

N3	7.256	6.229-8.283	
**Surgery**			0.006
No	6.988	6.415-7.561	
Yes	14.992	6.195-23.79	
**Radiation**			0.207
No	6.713	5.945-7.482	
Yes	7.230	6.475-7.986	
**Chemotherapy**			<0.001
No	3.862	3.357-4.367	
Yes	9.491	8.627-10.355	
