# Supplementary material for: Prognostic impact of metastatic patterns and treatment modalities on overall survival in lung squamous cell carcinoma: A population-based study
Source: Medicine (Baltimore). 2023 Jul 21;102(29):e34251. doi: 10.1097/MD.0000000000034251 (PMC10662909; doi:10.1097/MD.0000000000034251)
Supplement: Supplementary file 2 [file medi-102-e34251-s002.pdf]

Table S2 Multivariate survival analysis of patients with two metastatic sites.

Risk factors	HR	95% CI	*p*
**Metastasis site**			
Bone and brain metastasis	1.627	1.341-1.974	<0.001
Bone and liver metastasis	1.437	1.209-1.708	<0.001
Bone and lung metastasis	1.212	1.025-1.433	0.025
Brain and liver metastasis	1.780	1.393-2.275	<0.001
Brain and lung metastasis	1.233	1.011-1.504	0.039
Liver and lung metastasis	Ref		
**Age**			
＜65	Ref		
⩾65	1.137	0.974-1.078	0.146
**T stage**			
T1	Ref		
T2	1.221	0.966-1.544	0.095
T3	1.465	1.164-1.845	0.001
T4	1.429	1.139-1.791	0.002
**N stage**			
N0	Ref		
N1	1.098	0.893-1.349	0.376
N2	1.183	1.031-1.358	0.016
N3	1.137	0.973-1.329	0.107
**Surgery**			
No	Ref		
Yes	0.771	0.513-1.159	0.211
**Chemotherapy**			
No	Ref		
Yes	0.444	0.400-0.491	<0.001
