# Supplementary material for: Prognostic impact of metastatic patterns and treatment modalities on overall survival in lung squamous cell carcinoma: A population-based study
Source: Medicine (Baltimore). 2023 Jul 21;102(29):e34251. doi: 10.1097/MD.0000000000034251 (PMC10662909; doi:10.1097/MD.0000000000034251)
Supplement: Supplementary file 3 [file medi-102-e34251-s003.pdf]

Table S3 Univariate survival analysis of patients with three metastatic sites.

Risk factors	Mean of survival months	95% CI	*p*
**Metastasis site**			0.203
Bone and brain and liver metastasis	4.011	3.224-4.797	
Bone and liver and lung metastasis	7.056	5.097-9.016	
Bone and brain and lung metastasis	5.552	3.795-7.309	
Brain and liver and lung metastasis	4.078	2.832-5.325	
**Age**			0.011
＜65	7.043	5.159-8.928	
⩾65	4.835	3.775-5.894	
**Race**			0.560
White	5.586	4.516-6.656	
Black	4.915	3.321-6.508	
Others	8.409	1.943-14.875	
**Sex**			0.515
Female	6.831	4.475-9.186	
Male	5.181	4.244-6.119	
**Marriage**			0.200
Married	6.000	4.689-7.312	
Unmarried	4.740	3.782-5.698	
**Grade**			0.447
Well	2.667	1.360-3.973	
Moderate	7.138	3.599-10.678	
Poorly	5.210	3.625-6.795	
Undifferentiated	3.000	0.000-6.920	
Unknown	5.807	4.461-7.152	
**Primary Site**			0.612
Main bronchus	3.618	2.441-4.794	
Upper	5.372	4.237-6.507	
Middle	5.385	0.121-10.648	
Lower	5.925	3.929-7.921	
Others	8.097	3.719-12.476	
**T stage**			0.465
T1	7.778	3.943-11.612	
T2	4.392	3.478-5.305	
T3	5.489	3.701-7.277	
T4	6.266	4.644-7.888	
**N stage**			0.324
N0	4.603	2.267-6.940	
N1	4.700	2.861-6.539	
N2	5.310	4.129-6.490	
N3	6.130	4.566-7.694	

**Surgery**			0.773
No	5.763	4.747-6.778	
Yes	4.000	1.778-6.222	
**Radiation**			0.374
No	5.667	3.960-7.373	
Yes	5.757	4.546-6.968	
**Chemotherapy**			<0.001
No	2.632	1.900-3.365	
Yes	8.424	6.757-10.090	
